# Delayed cutaneous wound closure in HO-2 deficient mice despite normal HO-1 expression

**DOI:** 10.1111/jcmm.12389

**Published:** 2014-09-16

**Authors:** Ditte M S Lundvig, Alwin Scharstuhl, Niels A J Cremers, Sebastiaan W C Pennings, Jeroen te Paske, René van Rheden, Coby van Run-van Breda, Raymond F Regan, Frans G M Russel, Carine E Carels, Jaap C Maltha, Frank A D T G Wagener

**Affiliations:** a Department of Orthodontics and Craniofacial Biology, Radboud Institute for Molecular Life Sciences, Radboud University Medical CenterNijmegen, The Netherlands; b Department of Pharmacology and Toxicology, Radboud Institute for Molecular Life Sciences, Radboud University Medical CenterNijmegen, The Netherlands; c Department of Emergency Medicine, Thomas Jefferson UniversityPhiladelphia, PA, USA

**Keywords:** haem oxygenase, wound healing, skin

## Abstract

Impaired wound healing can lead to scarring, and aesthetical and functional problems. The cytoprotective haem oxygenase (HO) enzymes degrade haem into iron, biliverdin and carbon monoxide. HO-1 deficient mice suffer from chronic inflammatory stress and delayed cutaneous wound healing, while corneal wound healing in HO-2 deficient mice is impaired with exorbitant inflammation and absence of HO-1 expression. This study addresses the role of HO-2 in cutaneous excisional wound healing using HO-2 knockout (KO) mice. Here, we show that HO-2 deficiency also delays cutaneous wound closure compared to WT controls. In addition, we detected reduced collagen deposition and vessel density in the wounds of HO-2 KO mice compared to WT controls. Surprisingly, wound closure in HO-2 KO mice was accompanied by an inflammatory response comparable to WT mice. HO-1 induction in HO-2 deficient skin was also similar to WT controls and may explain this protection against exaggerated cutaneous inflammation but not the delayed wound closure. Proliferation and myofibroblast differentiation were similar in both two genotypes. Next, we screened for candidate genes to explain the observed delayed wound closure, and detected delayed gene and protein expression profiles of the chemokine (C-X-C) ligand-11 (CXCL-11) in wounds of HO-2 KO mice. Abnormal regulation of CXCL-11 has been linked to delayed wound healing and disturbed angiogenesis. However, whether aberrant CXCL-11 expression in HO-2 KO mice is caused by or is causing delayed wound healing needs to be further investigated.

## Introduction

Cutaneous wound repair occurs in temporally coordinated and overlapping phases: inflammation, granulation tissue formation and remodelling [1]. The timely progression of the different phases is coordinated by cytokines and growth factors, and each phase is characterized by the presence of specific cell types [2]. Both clinical and experimental studies have confirmed the importance for a well-regulated inflammatory resolution for proper wound healing, since prolonged inflammatory and oxidative stress may cause non-healing, chronic wounds or excessive scarring [3,4].

Haem oxygenases (HO) are enzymes that degrade haem into biliverdin, carbon monoxide and iron. Biliverdin is then converted into the antioxidant bilirubin by biliverdin reductase. HO-1 is highly inducible by a wide range of stresses, including inflammatory and oxidative stress, whereas HO-2 is mainly constitutively expressed [5,6]. The HO system is important in the resolution of inflammation [7,8]. The cytoprotective effects of the stress-induced HO-1 are evident in various pathological models and settings, whereas the constitutive HO-2 is important for physiological maintenance of the haem pool [9].

HO-1 deficient humans and mice demonstrate chronic inflammatory stress accompanied by increased leucocyte recruitment [10,11]. Moreover, genetic or pharmacologic ablation of HO-1 expression and activity in mice results in slower cutaneous wound closure [12]. Also, HO-2 deficient mice show delayed wound healing and an exaggerated inflammatory response after corneal epithelial wounding [13,14] which was associated with impaired HO-1 induction and function [13]. Notably, HO-2 levels have been suggested to regulate HO-1 expression and function in a cell and tissue-specific manner [15], and compensatory HO-1 expression in HO-2 deficient tissue has been reported [16,17]. Moreover, genetic or pharmacological HO-1 induction as well as administration of HO effector molecules biliverdin/bilirubin down-regulate the inflammatory response and restore wound healing in HO-1 deficient skin [12,18] and HO-2 deficient cornea [14,19]. On the other hand, HO-1 has pro-angiogenic effects *via* regulating VEGF synthesis [20,21]. Wound healing in HO-1 KO mice occurs with reduced neovascularization in the skin [12] whereas exaggerated angiogenesis was found in HO-2 deficient corneas [19]. Application of biliverdin ameliorated this pathologic angiogenesis occurring after corneal wounding in HO-2 KO mice [19].

Because of the described intricate involvement of both HO-1 and HO-2 in wound healing and regulation of inflammatory responses, this study focused on exploring the possible role of HO-2 in cutaneous wound healing using a full-thickness excisional wound model and HO-2 KO mice.

## Methods and materials

### Animals

Homozygote HO-2 KO mice generated by targeted disruption of the HO-2 gene [22] and WT mice were bred in-house on a mixed 129Sv x C57BL/6 background. Mice were provided with food and water *ad libitum* and maintained on a 12-hr light/dark cycle and specific pathogen-free conditions. All experiments and protocols were approved by the institutional Radboud University Nijmegen animal experimentation committee.

### Excisional wound model

Prior to wounding 6–12 weeks old female mice were shaved using an electrical clipper. The following day four full-thickness wounds were made on the back including the *panniculus carnosus* using a sterile disposable 4-mm biopsy punch (Kai Medical, Seki City, Japan) and the mice were left uncovered to heal (*n* = 18/genotype). At day 2 after wounding mice were killed, and the remaining mice (*n* = 9/genotype) were followed until day 7 (*n* = 9/genotype). For gene transcript analysis mice were wounded (*n* = 12/genotype) and killed at day 2 (*n* = 6/genotype) and day 5 (*n* = 6/genotype) after wounding. Punched out tissue at day 0 was collected as control. Wounds were collected using a disposable 6-mm biopsy punch (Kai Medical) allowing collection of the complete wound together with the surrounding normal tissue.

### Wound size analysis

Wounds were digitally documented on different days with a ruler placed next to the wounds for size normalization. Wound area was measured at least twice by a blinded investigator using ImageJ (NIH) v1.44p software.

### Immunohistochemistry

Tissue samples were fixed for 24 hrs in 4% paraformaldehyde and further processed for routine paraffin embedding. Sections were deparaffinized using Histosafe and rehydrated using an alcohol range. Endogenous peroxidase activity was quenched with 3% H_2_O_2_ in methanol for 20 min., and immunohistochemical stainings were performed and photographed as previously described [23]. Photographs were taken on a Carl Zeiss Imager Z.1 system (Carl Zeiss Microimaging Gmbh, Jena, Germany). For antibodies and antigen retrievals used, see Table 1.

**Table 1:** Antibodies used for immunohistochemistry

Antibody	Specificity	Dilution	Antigen retrieval	Source
SPA-895	HO-1	1:800	A	Stressgen
OSA-200	HO-2	1:800	A	Stressgen
MCA497R	F4/80	1:200	A	AbD Serotec, Kidlington, UK
2233PCO	Collagen IV	1:200	A	Euro-Diagnostica, Malmo, Sweden
Sc-34785	CXCL-11	1:100	B	Santa Cruz Biotechnology, Santa Cruz, CA, USA
A2547	αSMA	1:600	A	Sigma-Aldrich, St. Louis, MO, USA

A: 10 mM citrate buffer 70°C for 10 min, followed by trypsin digestion for 7 min.

B: 10 mM citrate buffer RT, 120 min.

### Semi-quantitative scoring of immunohistochemical sections

HO-1 and F4/80 immunoreactivity were evaluated as the number of positive cells (based on the percentage of positive staining) and staining intensity of four sections per animal. Extent was scored as: 0, ≤5%; 1, 6–25%; 2, 26–50%; 3, >50%. Intensity was scored as: 0, weak; 1, moderate; 2, strong. Intensity was designated as weak when only barely detectable. To correlate extent and intensity on the staining, a composite score was calculated by multiplying the individual scores of extent by intensity. Scoring was done three times by a blinded investigator. CXCL-11 immunoreactivity was evaluated by scoring a single section per animal by two independent investigators as previously described [24]. To assess αSMA immunoreactivity four sections per animal were scored twice as earlier described [25]. Vascularization was assessed twice on five high-power fields (HPF; 400× magnification) on four sections per animal as previously described [26]. A mean score per animal was used for further analysis.

### Collagen deposition

Collagen deposition in AZAN stained wound sections (1–4 sections/mouse) was determined by image analysis using a macro built in Image J [27]. The wound area was manually defined before running the macro using the edges of the *panniculus carnosus* and epithelium as boundaries. Measurements were performed twice, and mean intensity/mm^2^ per mouse was used for further analysis.

### Assessment of mRNA expression by quantitative real-time PCR

Total RNA was extracted from skin and wound samples by using Trizol (Invitrogen, Carlsbad, CA, USA) and a RNeasy Mini kit (Qiagen, Hilden, Germany), and cDNA was produced using the iScript cDNA synthesis kit (Bio-Rad, Hercules, CA, USA). To screen for differences in gene expression profiles of wound associated genes in HO-2 KO and WT mice, pooled cDNAs synthesized from individual wounds of HO-2 KO and WT mice isolated at day 5 were tested on a Mouse Wound Healing RT^2^ Profiler™ PCR Array according to the manufacturer's instructions (SABiosciences, Frederick, MD, USA). Individual cDNAs from all time-points were subsequently analysed for genes up- or down-regulated more than twofold using custom-designed primers (Table 2) and iQ SYBR Green Supermix (Invitrogen) in a CFX96 Real-Time PCR system (Bio-Rad). Relative gene expression values were evaluated using the 2^−ΔΔCt^ method using GAPDH as housekeeping gene [28]. Fold changes were normalized to WT mean day 0.

**Table 2:** Murine primers used for qPCR

Gene	Forward primer (5′→3′)	Reverse primer (5′→3′)	Reference
HO-1	CAACATTGAGCTGTTTGAGG	TGGTCTTTGTGTTCCTCTGTC	–
HO-2	AAGGAAGGGACCAAGGAAG	AGTGGTGGCCAGCTTAAATAG	–
TNF	CTCTTCTCATTCCTGCTTGTG	GAATTGTCCATCTGGCATAAC	–
CXCL-11	CACGCTGCTCAAGGCTTCCTTATG	TGTCGCAGCCGTTACTCGGGT	–
Gr-1	TGGACTCTCACAGAAGCAAAG	GCAGAGGTCTTCCTTCCAACA	–
F4/80	AATCCTGTGAAGATGTGG	GAGTGTTGATGCAAATGAAG	–
ACTA2	CAGGCATGGATGGCATCAATCAC	ACTCTAGCTGTGAAGTCAGTGTCG	[51]
GAPDH	GGCAAATTCAACGGCACA	GTTAGTGGGGTCTCGCTCCTG	–

### Western blotting

Protein was extracted from homogenized 4-mm skin biopsies in 100 μl lysis buffer [1 mM EDTA, 0.5% Triton X-100, Complete protease inhibitor cocktail (Roche, Penzberg, Germany), 100 μM PMSF], and Western blotting using primary antibodies against HO-1 and HO-2 (SPA-895 and OSA-200, 1:5000; Stressgen, Victoria, BC, USA) was performed as previously described [29].

### Statistical analysis

Statistical analysis was performed with GraphPad Prism 4.03 software. Normal data distribution were assessed using the Kolgomorov–Smirnov test. In case of non-normality, data were transformed. Statistical differences in qPCR data, wound closure and collagen deposition were determined using Student's *t*-test with Bonferroni-correction in case of multiple testing. Semi-quantitative scores were analysed with non-parametric Mann–Whitney test or Kruskall–Wallis test with Dunn's *post hoc* test. Collagen deposition, wound closure and qPCR data are presented as mean ± SD. Semi-quantitative scores are presented as box-and-whisker plots of median with 10–90 percentiles. *P* < 0.05 was considered statistically significant.

## Results

### Delayed cutaneous wound closure in HO-2 deficient mice

**Fig. 1:**
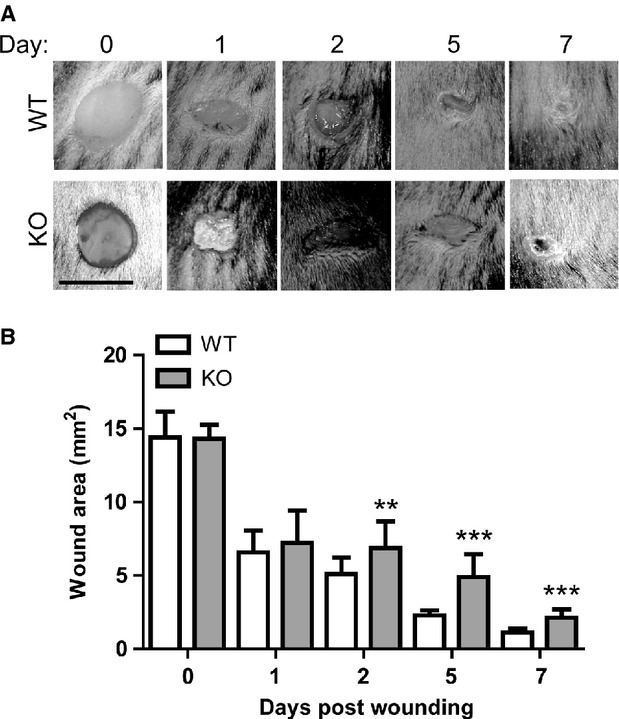
Slower cutaneous wound closure in HO-2 KO after excisional wounding. (**A**) Wound closure in WT (upper panel) and HO-2 KO (lower panel) mice in time; bar, 5 mm. (**B**) Wound area (mm^2^) reduction in WT (white bars) and HO-2 KO (grey bars) mice in time presented as mean ± SD. **P < 0.01, ***P < 0.001.

Prior to experimental start, we verified that HO-2 KO mice indeed were devoid of HO-2 mRNA and protein expression (Fig. S1). To investigate the role of HO-2 in cutaneous wound closure, full-thickness wounds were made on the backs of HO-2 KO and WT mice and monitored in time (Fig. 1). Wound area assessment demonstrated significantly larger wounds in HO-2 KO mice compared to WT controls at day 2 [6.9 ± 1.8 mm^2^ *versus* 5.1 ± 1.1 mm^2^ (*n* = 18/genotype); *P* < 0.01], day 5 [4.9 ± 1.6 mm^2^ *versus* 2.3 ± 0.4 mm^2^ (*n* = 9/genotype); *P* < 0.001] and day 7 [2.1 ± 0.6 mm^2^ *versus* 1.1 ± 0.3 mm^2^ (*n* = 9/genotype); *P* < 0.001] after wounding.

### Normal inflammatory response in HO-2 KO mice after cutaneous wounding

**Fig. 2:**
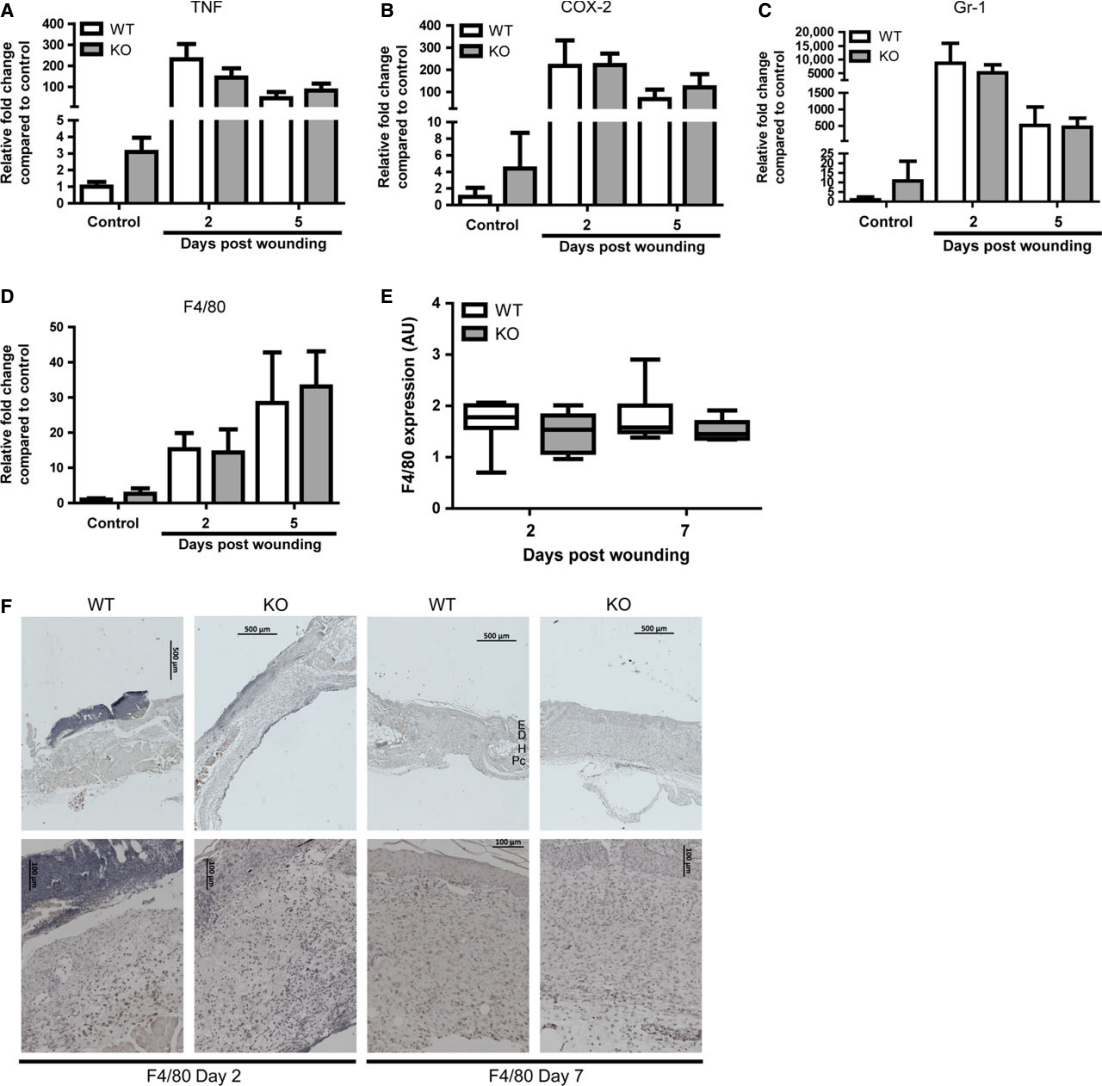
HO-2 KO mice demonstrate a normal inflammatory response after wounding. Gene transcript levels of pro-inflammatory proteins (**A**) TNF and (**B**) COX-2 and inflammatory cell markers (**C**) Gr-1, and (**D**) F4/80 in WT (white bars) and HO-2 KO (grey bars) mice in time presented as mean ± SD. Controls are tissue biopsies collected at day 0, and data were normalized to WT mean day 0. (**E**) Box-and-whisker plot with 10–90 percentiles of semi-quantitative assessment of F4/80 immunoreactivity in (**F**). (**F**) F4/80 immunoreactivity in representative wound sections of WT and HO-2 KO mice at day 2 and 7 after wounding. Anatomical indications by E, epidermis; D, dermis; H, hypodermis; Pc, panniculus carnosus; bars, 500 μm (upper panel), 100 μm (lower panel).

To determine whether delayed cutaneous wound healing in HO-2 KO mice is because of an exaggerated inflammatory response as observed after corneal injury, we compared gene expression profiles of different inflammatory factors and cell markers in skin of HO-2 KO and WT mice at day 2 and 5 (Fig. 2). The pro-inflammatory cytokine TNF and the stress-induced enzyme COX-2 demonstrated an injury-induced increase in transcript levels compared to unwounded skin in both WT and HO-2 KO mice, however, we did not detect any differences between the two genotypes at any of the investigated time-points (Fig. 2A and B). Furthermore, granulocyte marker receptor antigen-1, Gr-1 and macrophage marker F4/80 both demonstrated an injury-induced increase in transcript levels compared to unwounded skin, however, no differences between HO-2 KO and WT mice were evident (Fig. 2C and D). This was also reflected at the protein level, as semi-quantitative assessment of F4/80 immunoreactivity in wound sections demonstrated no significant differences between HO-2 KO and WT mice at day 2 and day 7 after wounding (Fig. 2E and F).

### Injury-induced HO-1 expression in both HO-2 KO and WT mice

**Fig. 3:**
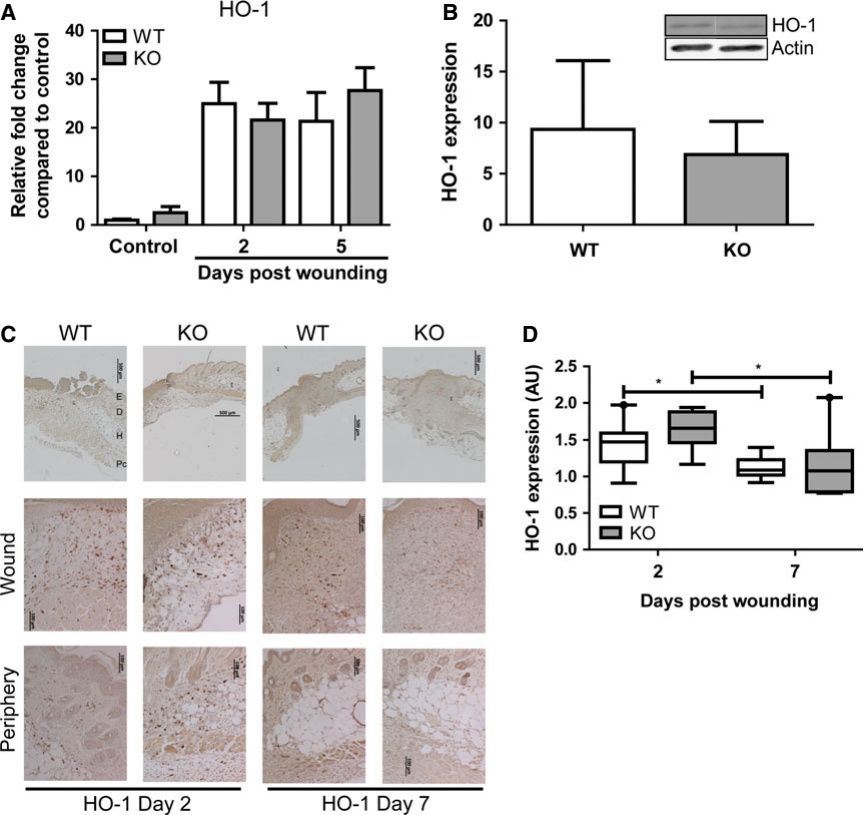
HO-2 KO mice induce cutaneous HO-1 expression after wounding. (**A**) HO-1 gene transcript levels in WT (white bars) and HO-2 KO (grey bars) mice in time as represented as mean ± SD. Controls represent biopsies collected at day 0, and data were normalized to WT mean day 0. (**B**) Western blot (insert) of cutaneous HO-1 expression in unwounded skin in WT (white bar) and HO-2 KO (grey bar) mice. Band intensity was normalized to housekeeping protein β-actin. Data are presented as mean ± SD. (**C**) HO-1 immunoreactivity in representative wound sections of WT and HO-2 KO mice at day 2 and day 7 after wounding. Anatomical indications by E, epidermis; D, dermis; H, hypodermis; Pc, panniculus carnosus; bars, 500 μm (upper panel), 100 μm (wound, periphery). (**D**) Semi-quantitative scores of HO-1 immunoreactivity in (**C**) presented as box-and-whisker plot with 10–90 percentiles. *P < 0.05.

In contrast to HO-2 deficient cornea [13] we detected both HO-1 mRNA transcript and protein in the skin of HO-2 KO mice (Fig. 3). Similar levels of HO-1 protein were detected in unwounded skin of HO-2 KO and WT mice (6.9 ± 3.2 and 9.3 ± 6.7, respectively; Fig. 3A). Also, a comparable injury-induced increase in HO-1 transcript levels was evident in both HO-2 KO and WT mice (Fig. 3B). Furthermore, injury-induced HO-1 protein expression was detected in both WT and HO-2 KO skin (Fig. 3C). HO-1 positive cells were present at high numbers both in the wound area and in the surrounding tissue at day 2, while at day 7 HO-1 positive cells were predominantly found in the wound area (Fig. 3C). Semi-quantitative assessment of HO-1 immunoreactivity demonstrated a significant reduction in HO-1 expression at day 7 compared to day 2 in both WT and HO-2 KO wounds (*P* < 0.05 for both genotypes), however, no significant differences between the genotypes were detected (Fig. 3D).

### Reduced collagen deposition and lower vessel density in HO-2 KO wounds

**Fig. 4:**
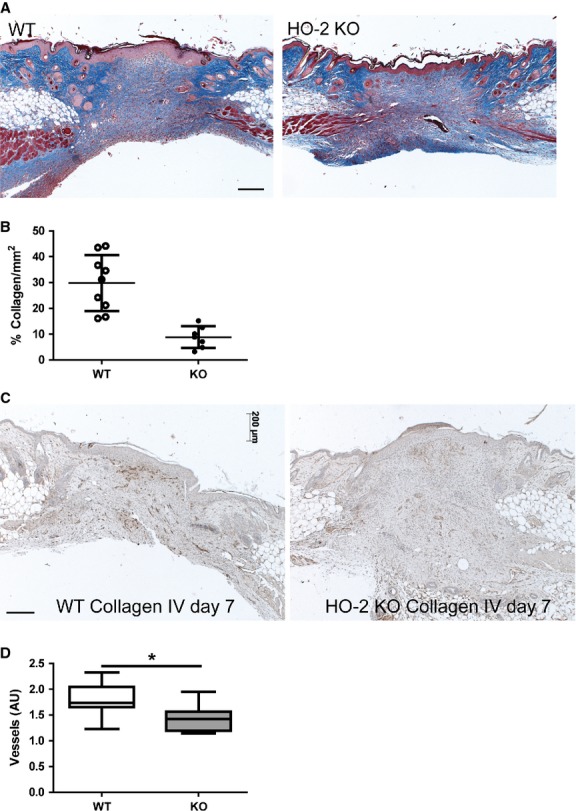
Reduced collagen deposition and vessel density in HO-2 KO mice. (**A**) Representative images of AZAN stained wound sections of WT and HO-2 KO mice at day 7 after wounding; bar, 200 μm. (**B**) Collagen deposition in WT (open circles) and HO-2 KO (closed circles) mice at day 7 after wounding. (**C**) Representative images of collagen IV immunoreactivity, a common blood vessel marker, in WT and HO-2 KO mice at day 7 after wounding; bar, 200 μm. (**D**) Semi-quantitative scoring of high-power fields of (**C**). Data are represented as box-and-whisker plot with 10–90 percentiles. *P < 0.05, ***P < 0.001.

HO-2 KO mice demonstrated no abnormalities compared to WT mice with respect to the inflammatory phase. We therefore questioned whether the delayed wound healing was a result of a dysregulated granulation phase. Assessment of collagen deposition in wound sections of HO-2 KO and WT mice using an Image J macro demonstrated a significant lower percentage collagen deposition per area in HO-2 KO wounds compared to WT controls at day 7 after wounding (8.8 ± 4.2%/mm^2^ *versus* 29.8 ± 10.8%/mm^2^, *P* < 0.001; Fig. 4A and B).

Also, we investigated the degree of vascularization in the wound areas of HO-2 KO and WT mice by semi-quantitative scoring of wound sections stained for collagen IV, a basement membrane protein found in the walls of blood vessels [30]. We detected a significant lower density of vessels per HPF in wound sections of HO-2 KO mice compared to WT mice at day 7 after wounding (*P* < 0.05; Fig. 4C and D).

**Fig. 5:**
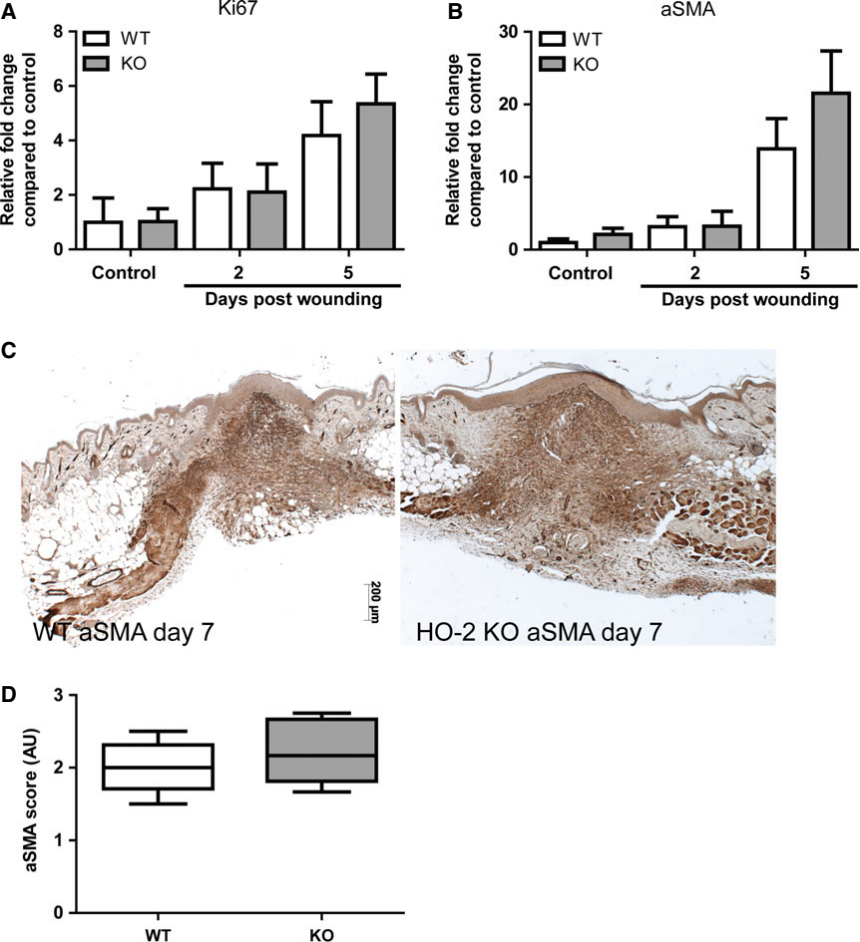
Myofibroblast differentiation occurs in HO-2 KO mice. Ki67 (**A**) and ACTA2 (**B**) gene transcript levels in WT (white bars) and HO-2 KO (grey bars) mice in time as represented as mean ± SD. Controls represent biopsies collected at day 0, and data were normalized to WT mean day 0. (**C**) Representative images of αSMA immunoreactivity in wounds of WT and HO-2 KO mice at day 7 after wounding; bar, 200 μm. (**D**) Semi-quantitative scoring of αSMA in (**C**) presented as box-and-whisker plot with 10–90 percentiles.

We wondered if the delayed wound healing could be related to a lower cell proliferation capacity in HO-2 deficient cells, and we therefore assessed the mRNA levels of Ki67, a marker of actively cycling cells [31]. We detected a time-dependent increase in Ki67 mRNA levels consistent with an increased influx and proliferation of wound repair associated cells; however, no differences between HO-2 KO and WT mice were evident at any of the investigated time-points (Fig. 5A).

During the granulation phase keratinocytes dominate epithelization and (myo)fibroblasts produce ECM such as collagen and close the wound [32]. We therefore investigated whether the presence of less (myo)fibroblasts could be an explanation for the reduced collagen deposition. We detected similar mRNA levels of ACTA2, the murine counterpart of myofibroblast marker αSMA, at all examined time-points (Fig. 5B). Moreover, we did not detect any difference in αSMA protein immunoreactivity level at day 7 after wounding as evaluated by semi-quantitative scoring (Fig. 5C and D).

### Different expression levels of CXCL-11 in WT and HO-2 KO mice after wounding

**Fig. 6:**
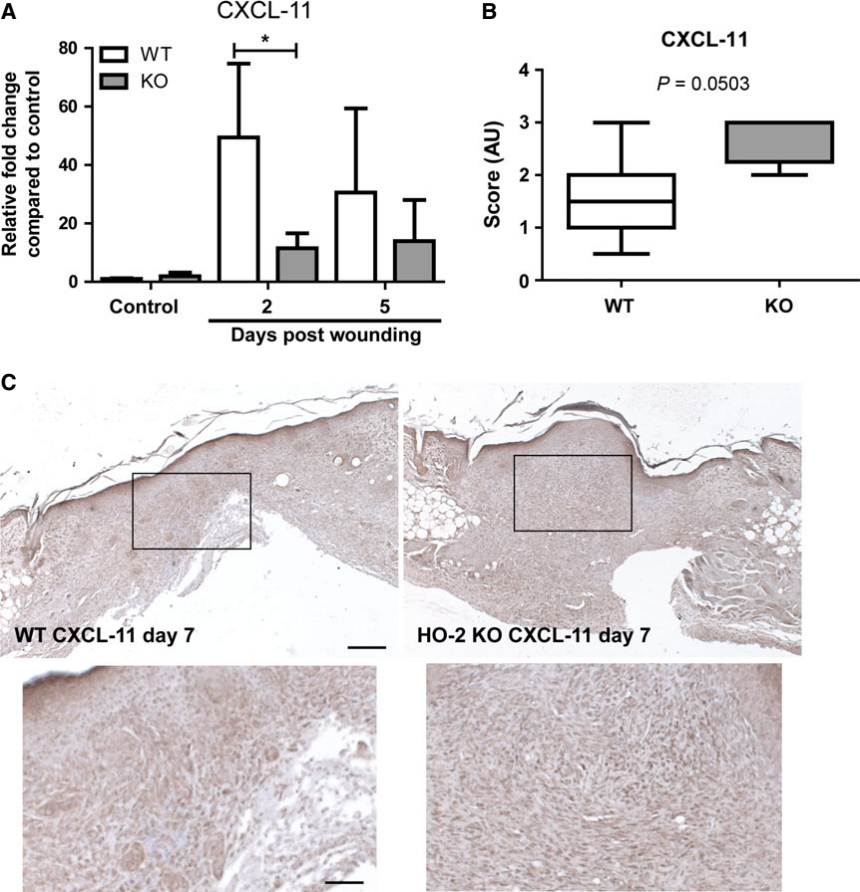
Different CXCL-11 expression in HO-2 KO and WT mice after injury. (**A**) CXCL-11 gene transcript levels in WT (white bars) and HO-2 KO (grey bars) mice in time presented as mean ± SD. Controls represent biopsies collected at day 0, and data were normalized to WT mean day 0. (**B**) Semi-quantitative scoring of CXCL-11 immunoreactivity in (**C**) of WT (white) and HO-2 KO (grey) wounds presented as box-and-whisker plot with 10–90 percentiles. *P < 0.05. (**C**) Representative images of CXCL-11 immunoreactivity in wound tissue of WT and HO-2 KO mice at day 7 after wounding; bar, 200 μm. Insert represent magnified boxed area; bar, 70 μm.

HO-1 deficiency is linked to delayed wound healing and impaired angiogenesis after injury. However, HO-2 deficient skin demonstrates injury-induced HO-1 expression, and we therefore wondered which genes could be explanatory for the reduced collagen deposition and lower vessel density observed in HO-2 KO mice. First we compared gene expression profiles of pooled cDNAs isolated from day 5 wound tissue of HO-2 KO and WT mice using a PCR wound healing array, followed by validation by custom-designed primers and individual cDNAs. Unexpectedly, after individual validation we only detected significant differences between HO-2 KO and WT mice in the expression profile of a single gene out of 84 screened genes, namely CXCL-11 (Fig. 6A, data not shown). Injury-induced CXCL-11 gene transcription observed in WT mice was absent in HO-2 KO mice at day 2 (49.4 ± 25.3 *versus* 11.5 ± 5.1; *P* < 0.05). However, at day 5 after wounding we detected similar CXCL-11 gene transcription levels in WT and HO-2 KO mice, which was mainly because of a down-regulation of gene transcription in WT mice (Fig. 6A). In contrast, immunohistochemical staining and semi-quantitative scoring of wound tissue isolated from HO-2 KO and WT mice 7 days after wounding showed a markedly higher level of CXCL-11 positive cells in the wounds of HO-2 KO mice compared to WT controls (*P* = 0.0503; Fig. 6B and C).

## Discussion

In this study, we investigated the role of HO-2 in cutaneous wound closure using HO-2 KO mice and a full-thickness excisional wound model. High levels of HO-2 in the skin have been suggested to be a first line of defense against acute injury [33]. Following excisional wounding we observed significantly slower cutaneous wound closure accompanied by reduced collagen deposition and lower vessel density in HO-2 KO mice compared to WT controls at day 7. The most pronounced difference in wound healing rates between HO-2 KO and WT seems to be during the first days of wound healing.

Cutaneous wounding is followed by haemolysis and free haem release leading to local oxidative stress and inflammation. HO-1 is rapidly induced after wounding [34,35] and promotes inflammatory resolution by down-regulating inflammatory mediators and attenuating infiltration of inflammatory cells [36,37]. Moreover, pharmacologic induction or overexpression of HO-1 accelerates both corneal and cutaneous wound healing [12,38]. On the contrary, HO-1 deficiency in man and mice leads to a chronic inflammatory state [10,11]. Also, pharmacologic or genetic inhibition of HO-1 slows down cutaneous wound closure *via* suppressed re-epithelization and neovascularization in murine models [12]. In HO-2 KO mice impaired corneal wound healing is associated with exaggerated inflammation [13,39]. We therefore have been suggested that also the delayed cutaneous wound closure observed in HO-2 KO mice results from an exaggerated inflammatory response.

Unexpectedly, we did not detect significant differences in the mRNA or protein expression level of the pro-inflammatory proteins, such as TNF and COX-2 and markers for granulocytes and macrophages. This rules out that exaggerated inflammation is the underlying cause of delayed cutaneous wound closure in HO-2 KO mice. The exaggerated corneal inflammatory response was associated with impaired HO-1 induction and function [13]. The importance of HO-activity was further supported by amelioration of corneal inflammatory resolution after application of biliverdin in HO-2 KO mice [14,19].

HO-2 can regulate HO-1 induction and function in a tissue and cell specific manner [15,17]. Increased HO-1 expression has been suggested to be a compensatory mechanism in HO-2 deficient lung and myocardium [16,40]. We detected similar levels of both HO-1 mRNA and protein expression in unwounded skin and injury-induced HO-1 up-regulation after wounding of HO-2 KO and WT mice, explaining the normal inflammatory resolution in both genotypes. Cell-type specific compensatory HO-1 expression likely dampens the inflammatory response after cutaneous wounding, as we observed a normal inflammatory phenotype in HO-1 expressing skin compared to the exaggerated inflammatory response in HO-1 deficient corneal tissue in HO-2 KO mice [13,39,41]. Notably, delayed cutaneous wound closure in HO-1 KO mice is also not accompanied by an uncontrolled inflammatory response following excisional wounding [12].

We next turned our focus to the granulation phase of cutaneous wound healing that is dominated by migration, proliferation and differentiation of fibroblasts, keratinocytes and endothelial cells in the wound area. Differential expression of HO-1 and HO-2 in keratinocytes and fibroblasts has been demonstrated [42]. This difference is critical for different sensitivities towards UV-induced oxidative stress [15,43]. This could also have consequences in a more complex setting, such as during wound repair. However, we did not observe any differences between HO-2 KO and WT mice with respect to the proliferation marker Ki67 or expression of the myofibroblast marker αSMA during the time course of wound healing.

Angiogenesis is a crucial process for proper wound healing, and disturbed blood vessel formation leads to delayed wound healing. Pro-angiogenic properties of HO-1 have been demonstrated [20,21]. Both diabetic db/db mice having weaker injury-induced HO-1 expression and HO-1 deficient mice demonstrate impaired vascularization and delayed wound closure which could be restored with HO-1 gene transfer [12]. Here, we also observed lower vessel density in the wounds of HO-2 KO mice despite normal HO-1 expression. This may restrict the blood supply to the healing tissue and set back wound healing.

To probe the molecular mechanism responsible for the delayed cutaneous wound closure in HO-2 KO mice, we used a PCR array containing 84 wound healing associated genes. Surprisingly, we only detected significant differences between HO-2 KO and WT mice in the expression profile of a single gene, namely CXCL-11. CXCL-11 is a versatile cytokine that *via* interaction with its receptor CXCR3 is thought to modulate several cell types important during several phases of cutaneous wound repair [44]. Decreased CXCL-11 or CXCR3 expression leads to delayed re-epithelization, impaired epidermis maturation and is associated with altered angiogenesis [45–48]. CXCL-11 is also an antagonist for C-C chemokine receptor type 5 (CCR5) [49]. CCR5 KO mice have delayed wound closure, impaired neovascularization and reduced collagen production following excisional wounding [50]. In contrast to WT controls, we did not detect any injury-induced up-regulation of CXCL-11 mRNA in HO-2 KO mice at day 2 after wounding. But, surprisingly, more CXCL-11 positive cells were present in the wounds of HO-2 KO mice compared to WT controls at day 7 after wounding. These observations imply that there is a slight delay in the expression of CXCL-11 gene and protein in HO-2 KO mice compared to WT controls. However, whether this is a consequence or a causing factor of the delayed cutaneous wound closure in HO-2 KO mice warrants further investigation. Summarizing, we demonstrated delayed dermal wound closure, decreased vascularization and reduced collagen deposition in HO-2 KO mice independent from inflammation and HO-1 expression. These data indicate a tissue-specific role for HO-2, as HO-2 seems to play a pivotal role in corneal, but not cutaneous, wound healing. This is directly linked to the regulation of injury-induced HO-1 expression. Furthermore, we report differences in the expression of the cytokine CXCL-11 between HO-2 KO and WT mice during wound repair. Whether this difference is causative or caused by the observed delay in cutaneous wound closure needs to be further investigated.

## Supporting information

**Figure S1** Cutaneous HO-2 expression in WT and HO-2 KO mice.
