# Supplementary material for: Delayed cutaneous wound closure in HO-2 deficient mice despite normal HO-1 expression
Source: J Cell Mol Med. 2014 Sep 16;18(12):2488–98. doi: 10.1111/jcmm.12389 (PMC4302653; doi:10.1111/jcmm.12389)
Supplement: Supplementary file 1 — Figure S1 Cutaneous HO-2 expression in WT and HO-2 KO mice. [file jcmm0018-2488-sd1.docx]

**Figure legends**

**Supplemental Figure S1. Cutaneous HO-2 expression in WT and HO-2 KO mice.**

(A) HO-2 gene transcript levels in WT (white bars) and HO-2 KO (grey bars) mice in time presented as mean ± SD. Controls represent samples collected at day 0, and data was normalized to WT mean day 0. (B) Western blot (insert) of cutaneous HO-2 protein expression in unwounded skin of WT (white bar) and HO-2 KO (grey bar) mice. Band intensity was normalized to housekeeping protein β-actin. Data is presented as mean ± SD. (C) Immunohistochemical staining of HO-2 in skin sections of WT and HO-2 KO mice at day 2 and day 7 after wounding. Anatomical indications by E, epidermis; D, dermis; H, hypodermis; Pc, panniculus carnosus. Bars, 500 µm (upper panel), 100 µm (lower panel).
