# Supplementary figures and images for: Delayed cutaneous wound closure in HO-2 deficient mice despite normal HO-1 expression
Source: J Cell Mol Med. 2014 Sep 16;18(12):2488–98. doi: 10.1111/jcmm.12389 (PMC4302653; doi:10.1111/jcmm.12389)

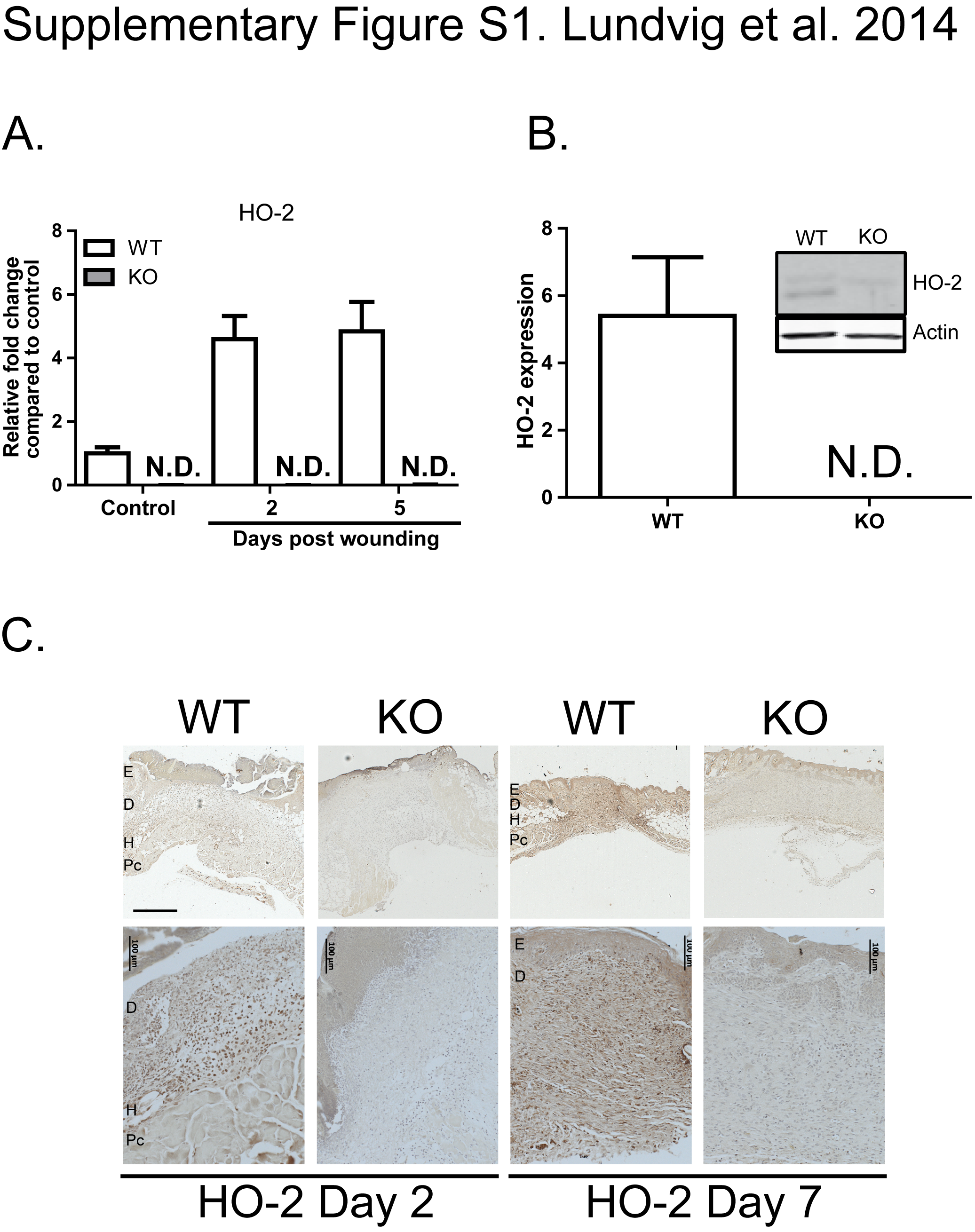

Supplement: Supplementary file 2 — Figure S1 Cutaneous HO-2 expression in WT and HO-2 KO mice. [file jcmm0018-2488-sd2.tif]
